# Glances at the Hospitals

**Published:** 1904-11-26

**Authors:** 


### GLANCES AT THE HOSPITALS.

### ROYAL INFIRMARY, EDINBURGH.

The ordinary income amounted to £32,685, and the ordinary expenditure to £47,600. Legacies and donations amounted to £47,000, and of that total £45,264 was given to the general fund, £900 to the extension fund, and £838 to the convalescent house fund. The infirmary has been added to, and new pavilions opened for the treatment of diseases of the eye, ear and throat, and the wards hitherto occupied by patients suffering from these diseases have been reappropriated. An electric medical department has been added, the skin-disease department has been improved, and additional beds provided for diseases of women. Statistics of nursing department show that the average number of nurses resident during the year was 210. There were 642 applications for admission, being 15 more than in the previous year. There were 60 resignations and 1 death among the nursing staff. At the beginning of the year the infirmary contained 683 patients, and 9,801 were admitted during it, making a total of 10,484 under treatment. Of these 4,921 were discharged cured, 3,480 were discharged relieved, 603 were discharged for various reasons, 722 remained under treatment, and 758 died. The average number of beds occupied was 743, and the average cost per bed was £64 1s. The average of each patient's stay in the hospital was nearly 26 days. The percentage of deaths to cases treated was 7·2; but if deaths occurring within 48 hours of admission be excluded the percentage would be reduced to 5·8. The medical and surgical tables are extremely well arranged, and show the age, sex, the numbers treated, the "cured," "relieved," "not relieved," "died," percentage of deaths and the stay in the wards. There were 97 cases of acute rheumatism, of which 79 were cured, 15 relieved, 1 not relieved, and 2 died. Of lobar pneumonia there were 200 cases, and of these 134 were cured, 5 relieved, 1 not relieved, 1 sent to surgical wards, and 59 died, showing a death-rate of 29·5 per cent. The total number of operations was 3,377, and the number of deaths therein was 188, showing a mortality of 5·56 per cent. There were 9 cases of suture for perforated ulcer of stomach, and death followed in 5 of these. One hundred and seventy-eight patients were operated on for the radical cure of hernia and apparently all were successful. There is no anæsthetic table printed, but we believe chloroform is almost the only agent used, still it would have been interesting and instructive to have a statement on the point.

### THE WALSALL HOSPITAL.

The receipts amounted to £3,471. The workpeople's own contributions reached the respectable figure of £1,390, and this was lower than that of the previous year. There is included in the general receipts a sum of £233, obtained partly from the nurses' home building fund, and we doubt whether this should be included in the income. The expenditure was under the income by nearly £50, a result of the year's working which is satisfactory to all concerned in the management. On January 1st the hospital contained 45 patients, and 865 were admitted, making a total of 910 under treatment. The average number of beds occupied was 50, and the average duration of each patient's residence was 17 days. Of the total number 791 were discharged cured or relieved, 12 were discharged not relieved, 64 died, and 43 remained in the wards. Except for the absence of an anæsthetic table the medical and surgical statistics are very creditably drawn out. Of fracture at the base of the skull there were 6 cases, with 3 recoveries and 3 deaths, and 8 cases of spinal fracture show 3 recoveries and 5 deaths. There were 12 operations for strangulated hernia resulting in 9 recoveries and 3 deaths. The total number of operations performed during the year was 412, being an increase of 152 over the previous year.

### BRISTOL GENERAL HOSPITAL.

The ordinary receipts amounted to £11,427, and the ordinary expenditure to £12,768, showing a deficiency of income of upwards of £1,340. It would seem that the subscriptions are only a little higher than in the previous year, and the donations would have been lower than usual had it not been for one sum of £1,000; but what is to be much regretted is that the workmen's own contributions have decreased by nearly £40. Among structural alterations has been the remodelling of the lavatory accommodation which cost nearly £3,000, and further outlay will be required for the provision of a second operating theatre for which plans have been approved and the work commenced. At the beginning of the year there were 167 patients in residence, and 2,094 were admitted during it, making a total of 2,261 under treatment during the year. Of these 1,234 were discharged cured, 619 were discharged relieved, 34 were discharged for other reasons, 208 died, and 166 remained in the wards. As regards the out-patient department we notice that over 36,000 cases were treated. The population of Bristol is about 225,000, so that something like one in every six received gratuitous medical advice at this hospital, and this is in addition to those who were attended in other institutions and by rate-paid medical men. An analysis of the more important medical and surgical cases is given. Of 34 cases of pneumonia 22 were cured and 12 died. There were 8 cases of ovarian cyst and these were all either cured or relieved. The tables are good as far as they go, but we miss a table of the operations and an anæsthetic table. As there are four house-surgeons or house-physicians there ought to be no difficulty in preparing these statistics. The secretary should also present a clear statement of the cost per head per week, the cost per patient, and the cost per bed occupied. The average of each patient's stay in the hospital should also be given.

